# Understanding Factors Influencing Polio Vaccine Uptake in Ghana—Developing Meaningful Community Mobilization and Engagement Strategies in Collaboration with Religious Leaders

**DOI:** 10.4269/ajtmh.22-0271

**Published:** 2022-10-31

**Authors:** Anna-Leena Lohiniva, Anastasiya Nurzhynska, Hudi Alhassan, Mrunal Shetye, Paul Ayiku

**Affiliations:** ^1^ UNICEF Ghana, Accra, Ghana;; ^2^ Viamo, Ghana Office, Accra, Ghana

## Abstract

This qualitative study explores how religious leaders in Ghana view polio and polio vaccine–related knowledge and perceptions of the community members. It also examines the personal characteristics of those who are most likely to accept or reject the vaccine. On the basis of the findings, this study provides a set of evidence-based recommendations to support religious leaders’ efforts to create polio vaccine demand in their communities. The study is based on focus group discussions conducted with religious leaders from various geographic locations across Ghana. The discussions were transcribed verbatim and analyzed thematically. Twenty religious leaders, including Christian, Muslim, and leaders of traditional African religions, participated in the study. The findings show that both religious leaders and community members lack knowledge and have multiple culturally and religiously influenced explanations for polio. In addition, the findings reveal that vaccine safety and efficacy are linked to emotional narratives, and receiving the polio vaccine is not a social norm in all communities. Educated mothers in urban settings were identified as those most receptive to the polio vaccine. To create polio vaccine demand, religious leaders need to combat misinformation and the negative perceptions about the vaccine. Recommendations include conveying high-quality information to community members, developing tactics to address culturally and religiously sensitive matters, using emotionally inspired personal accounts to enhance positive attitudes toward polio vaccines and act as catalysts for positive social norms towards the polio vaccine. Educated mothers from urban areas can be engaged as champions in vaccine demand creation.

## INTRODUCTION

The WHO Africa Region was certified free of wild poliovirus (WPV) in August 2020. However, in February 2022, Malawi declared an outbreak of wild polio, highlighting the need to focus on surveillance and vaccine demand creation as long as wild polio exists in the world.^1^ In addition, cases of circulating vaccine-derived poliovirus 2 (cVDPV2) continue to cause outbreaks in countries across Africa.^2^^,^^3^ Although Ghana has not experienced cVDPV2 outbreaks since 2021, the country remains vulnerable to reinfection and accordingly must continue initiatives to create vaccine demand.^4^

Community mobilization and engagement are key strategies to reduce vaccine hesitancy and to build trust in vaccination programs.^5^^–^^7^ Community leaders are crucial community mobilizers, particularly in countries where they are community gatekeepers with regard to access to the community and where they have an in-depth understanding of how the community perceives vaccines.^8^^–^^11^ Community leaders also play a central role in vaccine demand generation programming by contributing to local understanding about illness and health to ensure vaccine promotion activities are culturally relevant and acceptable.^12^ Lastly, community leaders can help improve trust in vaccines and vaccination programs by listening to the concerns within the community—in particular, those who have refused to participate in polio eradication programs, to ensure programming and messaging strategically addresses their concerns.^13^ There are a number of examples of successful engagement of community leaders in polio vaccine demand generation programs. In Nigeria, the success of the entire polio program is credited to their community mobilization and engagement efforts in which community leaders played a major role.^14^ The engagement of religious leaders as community mobilizers has shown to be an effective strategy to improve vaccine uptake in many countries where they are respected and trusted such as in India, Indonesia, Nigeria, and Pakistan.^15^^–^^17^ Religious leaders are also known to play an important role in vaccine demand creation as they can help improve the competence, autonomy, and belonging of vulnerable communities with regard to vaccine demand generation as they usually have access to these communities.^18^^,^^19^

In Ghanaian society, respect for the elderly is expected and the traditional institution of community leaders is appreciated.^20^^,^^21^ The country is extremely diverse with more than 70 ethnic groups and more than 500 languages spoken, reflecting the diversity of the religious leaders, which include (Christian, Muslim, and traditional African religions). Religious leaders are often seen as informal liaisons between local communities and state institutions, which makes them ideal community mobilizers in both rural and urban areas in Ghana. Their role extends to political, economic, educational, religious, and family life areas. They understand and shape concepts of illness, health, and childcare in the Ghanaian culture, which in turn, influences health-seeking behaviors including vaccine uptake.^21^^–^^24^ Therefore, it is of utmost importance to engage them in polio vaccine demand creation strategies. Involving religious leaders in health promotion is also beneficial because large-scale gatherings allow them to fulfill their traditional stewardship roles as leaders and protectors of the community.^25^ Religious leaders in Ghana are commonly involved in the planning phase of community development activities.^24^ For example, UNICEF has successfully engaged Ghanaian community leaders in health, education, and child protection programs such as urban sanitation and hygiene, education, routine immunization, and others.

To develop community engagement and mobilization programming for polio vaccine demand, there is a need to understand what drives it. Knowledge is rarely a trigger for changing behaviors. Instead, behaviors are influenced by various psychological, social, and environmental factors that must be understood in a given context. These insights can be used to develop evidence-based interventions.^26^ Human-centered interventions are commonly used to develop context-specific approaches based on the “users,” which ensures that messages and tools are tailored and context-specific. Human-centered approaches are based on research rather than assumptions of the program designers.^27^

This study follows human-centered approaches by engaging religious leaders in vaccine demand creation by exploring their understanding of community perceptions about polio and the vaccine. The findings are used to identify strategies and interventions on how to support religious leaders’ efforts to conduct polio vaccine demand activities within their communities. The proposed strategies and interventions will be shared with religious leaders for their verification.

## METHODOLOGY

This qualitative study uses focus group discussions (FGDs) as data collection tools. The study follows the Behavioral Drivers Model by exploring the factors that influence polio vaccine demand and the type of community members more likely to accept the polio vaccine^28^ as such individuals can be engaged by religious leaders as catalysts for vaccine demand. Accordingly, a semi-structured question guide was developed that included questions covering psychological, social, and environmental factors. The questions were discussed with a number of experts, piloted, and adjusted accordingly.

The sampling was purposive, based on maximum variation^29^ and included male and female leaders from different regions and from both urban and rural areas. Recruitment was based on snowballing, where the study coordinator contacted religious associations with whom UNICEF collaborates and asked them to nominate religious leaders who may be interested and willing to participate in the study. The study coordinator called each nominated leader, explained the study’s purpose, and invited them to join a FGD in the capital city of Accra. If the leader was willing to join, the coordinator sent them an invitation letter that stated the place and the time of the FGD. Leaders who were traveling from distant regions were offered accommodation.

The FGDs were conducted by four research team members who were experienced in collecting qualitative data. All of them were Ghanaians who were accustomed to discussing issues with religious leaders. The team had a 1-day training during which they learned about the aims and the objectives of the study and familiarized themselves with the question guide. Each group had a moderator, a note-taker, and a translator. As the majority of the FGD participants spoke English, the FGDs were conducted in English, but with a professional translator for the few participants who only spoke Twi. The FGDs lasted from 2 to 3 hours with a break in between. The FGDs started with an introduction and informed consent that followed the signing of written consent. The question guide included 10 questions with several probes. The FGDs were audio-recorded and transcribed. Transcripts in Twi were translated into English. One team member carried out a thematic analysis^31^ that included familiarization with the material by reading the transcript multiple times followed by inductive coding that was based on identifying psychological, social, and environmental factors. The codes with corresponding quotes were organized in a spreadsheet and then collapsed into categories that lead to themes. The second team member discussed the coding, categorization, and themes that lead to the final set of themes and the interpretation of the findings in consensus with all four of the research team members. The study received ethical approval by the Ghana Health Services ethical review board. All participants signed written consent forms. No identifiers were collected from the study participants during the study, and confidentiality for all data was maintained during and after the study.

## FINDINGS

### Demographics of the study participants.

The sample included a total of 20 religious leaders from 11 of the 16 regions of Ghana. They included 10 Christian religious leaders, eight Islamic religious leaders, and one male and one female who were leaders in African traditional religions. More than half the respondents (12 of 20) were 41 to 60 years old with the remaining either over age 61 or 21 to 40 years old. Almost half of the participants came from rural areas (7 of 20).

### Factors influencing uptake of the polio vaccine.

#### Perceptions of polio.

Most respondents believed that people in their communities had heard of polio. However, they believed that people had different understandings about the causes of polio. Some respondents pointed out that polio was caused by a virus, and they believed that people in their communities thought likewise. Others, however, cited that people commonly believed that polio was caused by an ancestral curse, which is perceived a serious matter involving not only the person with polio but the entire family and maybe even the tribe. Other explanations for the causes of polio included contaminated water or the environment.

Some respondents highlighted that some in their communities believed that polio was curable whereas other believed the opposite, but a number of people were also unsure if a cure existed.

I don’t know the causes of polio. So it is hard for people who have not been to school to also know the causes of polio. *(A female Christian leader)*

Several respondents firmly stated that polio resulted in paralysis and even death, which they believed was known in the communities. Some respondents pointed out that whenever people had heard or seen those with side effects, such as paralysis, they were more likely to perceive polio as threatening

Even though we have not seen anyone die from polio, we think it can cause death. *(A traditional male leader)*

#### Perceived efficacy of the polio vaccine.

Many respondents highlighted that community members had doubts about the ability of the vaccine to prevent children from getting polio. They noted that people commonly believed that many children contracted polio despite having received the vaccine. They cited having witnessed polio among vaccinated children or having heard of such cases in the community. Some respondents themselves claimed to have seen polio cases among vaccinated children.

I have seen it. I have seen children that have taken the vaccine but were still paralyzed! *(A female Muslim leader)*

Others explained that community members believed poliovirus was strong and able to survive dormant in people for long periods of time making the virus difficult to control with a vaccine. Some respondents clarified that community members had deep-rooted beliefs that true immunity against diseases was only achieved by contracting the virus.

I think the vaccine is not enough for the children. This is because even after taking the vaccine, some of the children still get polio. *(A female Muslim leader)*

#### The perceived safety of the polio vaccine.

Most respondents believed that community members had doubts about the safety of the polio vaccine. Several respondents shared minor incidents of adverse events after immunizations that they heard took place in their communities, which elevated concern about immunization in general, but also about the polio vaccine. Often, respondents were not aware of the details or reason for these incidents but raised it as a concern.

They injected the boy’s hand, and the hand got deformed. I don’t know what happened. *(A female Muslim leader)*

One respondent explained that some people believed that the polio vaccine could kill. Another respondent shared that someone had been paralyzed after receiving the polio vaccine. Overall, respondents agreed that incidents of any adverse events created rumors that reduced community members’ interest in giving the polio vaccine to their children. Participants also noted past concerns about infertility being linked with the polio vaccine which are still circulating in various communities. Some respondents clarified that parents whose children had reactions to immunization in the past were usually those who did not want to take any immunizations including the polio vaccine.

In addition, some respondents had heard of vaccine-derived polio, which made them cautious. One respondent highlighted that polio eradication has been effective, but because the vaccine caused the current polio wave in the country, he no longer thinks the vaccine is safe. Respondents requested to learn more about vaccine-derived polio.

We have been able to eradicate polio for a while, so based on that we can say it is effective. However, this wave of polio is said to be vaccine-derived. This raises the question as to whether or not the vaccine is actually safe. *(A male Christian leader)*

#### Indifferent attitude of parents toward the polio vaccine.

Respondents also highlighted that not all parents consider the polio vaccine important, and accordingly they do not necessarily give all the doses of the vaccine to their children or they give the vaccine late, which could partly explain why even vaccinated children developed polio.

You see, sometimes parents have a problem. They will take their children for the first dose but skip the second and third dose. So I get your concern. *(A male Muslim leader)*

#### Mistrust toward modern health systems.

Some respondents explained that people who do not rely on modern healthcare systems but use traditional healers, especially those who use traditional birth attendants, usually do not believe in immunization or any other modern pharmaceutical interventions. Some respondents clarified that mothers who used traditional healers were usually less exposed to mother and child health messages, including immunization as they delivered their children using traditional birth attendants.

Those are the people who do not deliver in the hospital. They have nothing to do in the hospital. They don’t go for antenatal care, they do nothing. *(A female Muslim leader)*

### Religious beliefs.

Respondents stated that religious beliefs also played a critical role in whether people accept or reject polio vaccination. Some of them explained that there were some faith-based groups that believed only in the healing power of God and disregarded modern medicines, including vaccines, as a means to protect one from disease. A few respondents also pointed out that certain Christian denominations such as Jehovah Witness and Seventh Day Adventist church members may refuse vaccines as needles involve blood. Others explained that in some villages traditional healers rejected vaccinations as a healing remedy as they believe solely in traditional herbal treatments, which they also convey to their clients. Respondents also noted that similar beliefs in the use of traditional herbal remedies over modern pharmaceuticals can be found in other ethnic groups such as Nomads coming from Nigeria.

Faith-based communities believe that they are not to use medicine when they are sick. *(A male Christian leader)*

### Personal characteristics of those more likely to accept or reject the polio vaccine.

#### Educational level, age, and urban residency.

Several respondents pointed out that educated people were more likely to take the vaccine in their communities because they had more information about the benefits of the vaccine. They also noted that women were often better informed than men about child health because they receive information on polio and polio immunization at the health centers during routine immunization visits. One respondent highlighted that educated people who were able to evaluate and judge what they heard were in favor of immunization including the polio vaccine. A few respondents also cited that urban residents accept the vaccine more than rural residents do as they are more exposed to information. However, others believed that rural residents have the time to engage in healthcare including taking their children for vaccines as the rural dwellers have fewer formal engagements compared with their urban counterparts due their level of education. Respondents also believed that young teenage mothers were less likely to vaccine their children as they were uninterested and careless toward their infants.

## DISCUSSIONS

This study provides important insights about the knowledge and perceptions of community members in Ghana conveyed by religious leaders regarding polio and the polio vaccine. On the basis of the findings, a set of recommendations have been developed to better support religious leaders in their polio vaccine demand creation activities. The study aligns with GPEI strategies to undertake evidence-based polio vaccine promotion and to place human behavior in the center to fight polio.^32^^,^^33^

The findings indicate that both religious leaders and community members are confused about the causes and the severity of polio infection, which are similar to findings in Nigeria and Pakistan.^34^ This highlights the need to provide high-quality information about polio to religious leaders and to shed light on how they can deliver this information to community members. Lessons learned from the Polio Communication Network (PCN), which was established to respond to the polio outbreaks in Horn of Africa, could be used in Ghana by involving religious leaders in microplanning how information can be shared at the community level and the type of information needed to curb information voids.^35^

The findings also indicate that community members have pluralistic illness explanations of polio. For example, some believe that polio is caused by a virus, whereas others believe is a result of an ancestral curse, highlighting the cultural and religious explanations that require consideration. When the cause of the illness is believed to be due to spirits and ancestors, community members are likely to seek care from traditional healers.^36^ This means that biomedical explanations and messaging are not understandable or acceptable for all. Ghana can benefit from previous studies and lessons learned to address concepts linked with the local culture and religions. Studies in Pakistan suggest breaking cultural barriers by communicating ethical principles.^37^^,^^38^ PCN on the other hand has developed standard messages for polio and child survival that are reinforced by religious teachings from the Bible and Quran.^35^ Religious leaders can also learn to use specific communication frameworks to manage cultural and religious concepts that people link with polio and the vaccine such as LEARN, which is a process including listening, explaining, acknowledging, recommending, and negotiating that builds on culturally competent communication. It is based on a nonjudgmental approach that builds trust between those who communicate biomedical ideas and those whose worldview also includes alternative explanations.^39^ In South Sudan, healers have been sucessfully sensitized on the importance of the polio vaccine and engaged in community mobilization activities.^40^ Overall, it is important that religious leaders are given an opportunity to consider various cultural and traditional aspects to determine appropriate vaccine demand creation strategies and messages to use when creating polio vaccine demand in their communities.

Lack of standard knowledge of polio and the polio vaccine as well as cultural influences can also reduce the perceived risks related to polio and increase the perceived risks related to the polio vaccine, which is likely to increase vaccine hesitancy.^41^ However, studies also indicate that although risk perception may prompt behavior during a crisis situation, the impact is not necessarily long-lasting. Accordingly, efforts to increase risk perception of polio may not be the most relevant way to create long-term vaccine demand for polio.^42^ It is of utmost importance that community leaders have sufficient knowledge about the risks of polio and the safety and risks of the polio vaccine to feel confident engaging communities on the topic of vaccine demand. Moreover, information voids are likely to increase mistrust toward the vaccine and vaccination programs and increase the spread of misinformation.^43^

The study also indicates that both religious leaders and community members have doubts about polio vaccine efficacy and safety, which have also been identified as key factors influencing vaccine hesitancy among parents in India, Nigeria, and Pakistan.^44^^–^^46^ The doubts are based on personal and emotional accounts shared in the communities, which makes them powerful and difficult to mitigate. Moreover, misleading information that spreads quickly and widely often appeals to emotion.^47^ Accordingly, there is a need to build the capacity of community leaders to deal with emotional reactions based on misinformation linked to personal stories about the efficacy and safety of the vaccine. Therefore, religious leaders also require capacity building on how to better identify and manage misinformation with some basic techniques such as debunking and prebunking.^48^^,^^49^ They also need capacity building on how to respond to emotional narratives that may include techniques such as the use of therapeutic storytelling that has shown to impact emotions.^50^ Community leaders have the advantage of having the same worldviews as the community members, which can facilitate the communication and trust building that is required to shift false perceptions about the polio vaccine.^47^^,^^51^ It would also be worthwhile to familiarize community leaders with the basics of social and behavioral communication including the cognitive biases that are likely to influence community members’ acceptance of polio efficacy and safety-related messaging, such as anchoring bias in which people strongly influenced by information that they heard before or simplicity bias in which complex information has been generalized leading to misinformation.^47^^,^^51^

Parents’ indifferent attitudes toward the polio vaccine indicate a lack of motivation as in several studies in other parts of the world.^52^ Accordingly, it would be important to engage community leaders and community members jointly to identify motivational factors that are culture and context specific and to design motivational strategies and tools that can be used in vaccine demand generation. The attitude of the parents also highlights that the polio vaccine is not a norm in all communities. Religious leaders can be used as catalysts for changing community norms regarding the polio vaccine by focusing efforts on positive behavior change towards the vaccine. In Pakistan, vaccinators were used as catalysts to promote positive social norms toward the polio vaccine.^53^

Educated women in urban areas are believed to be more likely to accept the polio vaccine than others. Community outreach activities can focus on those women and engage them as champions and role models for polio vaccine demand generation.^40^ The use of “champions” is known to be an effective way to change community norms.^40^ In addition, community outreach efforts could also focus on those who are more reluctant to take the vaccine by using community leaders’ in-depth understanding about the reasons for their reluctance and developing targeted and personalized messages to address them.^54^

The study includes in-depth insights into community perceptions about polio and the polio vaccine. However, the study had some limitations. There may have been some social desirability bias because the groups of community leaders were strongly diverse with different values and beliefs that may have influenced how they answered questions in the focus group discussions. For example, respondents may have tried to avoid any conflict or confrontations by not sharing their true opinions about religious, political, or socioeconomic barriers to immunization including infrastructural challenges or discrepancies in vaccine coverage that have been identified by GPEI in other countries such as in India, Nigeria, and Pakistan.^55^ It is also important to be careful with generalization of the study findings to all Ghanian communities because the sampling of the study was purposive. However, to mitigate this weakness, the study’s sampling plan was carefully designed using a maximum variation to provide a diverse range of religious leaders to provide as much insight as possible on the topics discussed.

## CONCLUSIONS

The study resulted in a set of recommendations to support religious leaders in polio vaccine demand creation. They include the provision of high-quality information and tools to convey this information to community members and the provision of tools to communicate biomedical information to people with culturally and religiously diverse illness explanations, capacity building to identify and manage misinformation, the provision of communication skills and tools to address emotional narratives, a basic understanding of social and behavioral change communication, and focused efforts to enhance positive attitudes of religious leaders toward polio vaccines to act as a catalyst for social norm change around polio vaccines in their communities.
